# Isotope dependence of the Zeeman effect in lithium-like calcium

**DOI:** 10.1038/ncomms10246

**Published:** 2016-01-18

**Authors:** Florian Köhler, Klaus Blaum, Michael Block, Stanislav Chenmarev, Sergey Eliseev, Dmitry A. Glazov, Mikhail Goncharov, Jiamin Hou, Anke Kracke, Dmitri A. Nesterenko, Yuri N. Novikov, Wolfgang Quint, Enrique Minaya Ramirez, Vladimir M. Shabaev, Sven Sturm, Andrey V. Volotka, Günter Werth

**Affiliations:** 1 Atomic Physics Division and Superheavy Element Physics Division, GSI Helmholtzzentrum für Schwerionenforschung, Planckstraße 1, 64291 Darmstadt, Germany; 2 Stored and Cooled Ions Division, Max-Planck-Institut für Kernphysik, Saupfercheckweg 1, 69117 Heidelberg, Germany; 3 Superheavy Element Physics Division, Helmholtz-Institut Mainz, Johann-Joachim Becherweg 36, 55128 Mainz, Germany; 4 Institut für Kernchemie, Johannes Gutenberg-Universität, Fritz Strassmann Weg 2, 55128 Mainz, Germany; 5 Department of Physics, St Petersburg State University, Ulianovskaya 1, Petrodvorets, St Petersburg 198504, Russia; 6 Department of Physics, Institut für Theoretische Physik, Technische Universität Dresden, Mommsenstraße 13, 01062 Dresden, Germany; 7 Institute for Theoretical and Experimental Physics, Kurchatov Institute, B. Cheremushkinskaya street 25, Moscow 117218, Russia; 8 Petersburg Nuclear Physics Institute, Gatchina, 188300 St Petersburg, Russia; 9 Institut für Physik, Johannes Gutenberg-Universität, Staudingerweg 7, 55128 Mainz, Germany

## Abstract

The magnetic moment *μ* of a bound electron, generally expressed by the *g*-factor *μ*=−*g μ*_B_ *s ħ*^−1^ with *μ*_B_ the Bohr magneton and *s* the electron's spin, can be calculated by bound-state quantum electrodynamics (BS-QED) to very high precision. The recent ultra-precise experiment on hydrogen-like silicon determined this value to eleven significant digits, and thus allowed to rigorously probe the validity of BS-QED. Yet, the investigation of one of the most interesting contribution to the *g*-factor, the relativistic interaction between electron and nucleus, is limited by our knowledge of BS-QED effects. By comparing the *g*-factors of two isotopes, it is possible to cancel most of these contributions and sensitively probe nuclear effects. Here, we present calculations and experiments on the isotope dependence of the Zeeman effect in lithium-like calcium ions. The good agreement between the theoretical predicted recoil contribution and the high-precision *g*-factor measurements paves the way for a new generation of BS-QED tests.

## Abstract (web summary)

In addition to hyperfine splitting effects, isotope shifts of atomic electronic energy levels allow the investigation nuclear properties. Here, the authors study the isotope dependence of the Zeeman effect in litihium-like calcium isotopes in a Penning-trap setup and find good agreement with QED calculations.

## Graphical abstract

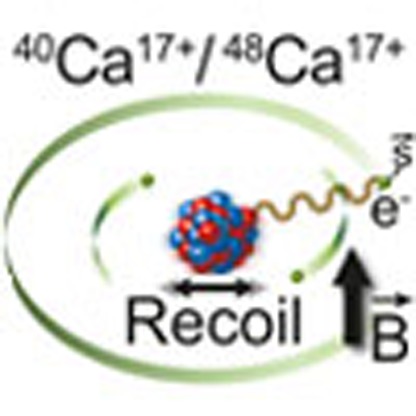

Besides hyperfine splitting, isotope shifts of atomic electronic energy levels provide the most common access to nuclear properties^1^. Typically, the dominating nuclear effects contributing to isotope shifts are generated by differences in nuclear masses, also denoted as nuclear recoil shifts (mass shifts), and by differences in nuclear sizes due to different spatial distributions of the nuclear charge (field shift). In absence of the magnetic field, isotope shifts in highly charged ions were first measured in refs 2, 3. In particular, relativistic nuclear recoil shifts have been previously probed in experiments on the isotope shifts in the binding energy of boron-like argon^4^ and lithium-like neodymium^5^.

As already proposed for different magnesium isotopes^6^, in this paper, we focus on the isotope dependence of the Zeeman effect by studying *g*-factors of lithium-like calcium isotopes ^40^Ca^17+^ and ^48^Ca^17+^. Featuring on the one hand a 20% mass difference and on the other hand almost identical nuclear charge radii^7^, these isotopes provide a unique system across the entire nuclear chart to test the relativistic nuclear recoil shift in presence of a magnetic field.

Most physical effects contributing to *g*-factors of highly charged ions, for example, the relativistic, radiative, nuclear size or interelectronic-interaction corrections, are calculated using bound-state quantum electrodynamics (QED) in the infinite-nuclear-mass approximation. Here, the nucleus is considered as an external Coulomb potential fixed in space. This approach is usually denominated as the Furry picture of QED (ref. 8). However, bound-state QED contributions of the studied nuclear recoil shift require calculations beyond the Furry picture, which are presented in the first part of this paper.

The experimental determination of the tiny *g*-factor difference Δ*g*≡*g*(^40^Ca^17+^)−*g*(^48^Ca^17+^), which is in the order of 1 × 10^−8^, requires four independent high-precision measurements: the Larmor-to-cyclotron frequency ratios of both calcium ion species as well as their atomic masses. The frequency ratios have been measured successively with a relative uncertainty of about 7 × 10^−11^. For this purpose, we studied single ions confined in a dedicated Penning-trap set-up^9^,^10^. Aiming for atomic masses with relative uncertainties of about 4 × 10^−10^, we also improved the atomic mass of ^48^Ca by a factor of seven. Here, we used the offline configuration of the Penning-trap mass spectrometer SHIPTRAP (ref. 11) in combination with the novel phase-imaging ion-cyclotron resonance technique (PI-ICR)^12^,^13^. The finally obtained 1.0*σ* agreement between the predicted and measured *g*-factor difference decisively confirms relativistic recoil corrections in the presence of strong fields. The reinforced understanding of the interaction between the bound electrons and the nucleus provides the opportunity to extract fundamental constants, namely the fine structure constant *α*, and nuclear properties via *g*-factor measurements in heavy atomic systems^14^.

## Results

### Calculation of the *g*-factor difference

The theoretical value of the isotope shift in the atomic *g*-factors is mainly given by a sum of the nuclear recoil and nuclear size contributions. Considering *s*-states of highly charged ions, the leading order terms scale with 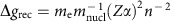 and 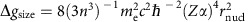 (ref. 15), where *n* represents the principle quantum number of the valence electron. Further nuclear contributions, for example, nuclear deformation^16^ and nuclear polarization^17^ are orders of magnitude smaller and at the current level of experimental as well as theoretical precision extraneous to the *g*-factor difference. For *Z*=20 the isotope shift is essentially determined by the mass shift, which in the case of *s*-states is of pure relativistic origin. Considering the two double magic isotopes ^40^Ca and ^48^Ca, the nuclear charge radii *r*_nucl_(^40^Ca)=3.4776 (19) fm and *r*_nucl_(^48^Ca)=3.4771 (20) fm (ref. 7) are surprisingly similar and by itself subject of present research. In this way, the nuclear recoil shift dominates the *g*-factor difference of the lithium-like electron configuration to 99.96%.

The lowest order recoil correction, which is non-QED but relativistic, can be derived from Breit equation^18^,^19^,^20^,^21^. The full relativistic theory of the nuclear recoil effect on the atomic *g*-factor has to be formulated in the framework of QED. So far, a systematic approach has been developed to first order in the electron to nucleus mass ratio *m*_e_·*m*_nucl_^−1^ and to all orders in *Zα* (ref. 22). As a result, the complete *Zα*-dependence formula for the recoil effect on the *g*-factor of a hydrogen-like ion has been derived. To zeroth order in *Z*^−1^, this formula describes also the recoil effect in a few-electron ion with one electron over closed shells, provided the electron propagators are defined for the vacuum with the closed shells included^15^. Generally, this leads to the appearance of two-electron nuclear recoil contributions. However, for the (1*s*)^2^2*s*-state of a lithium-like ion, the two-electron contributions vanish, and, therefore, to zeroth order in *Z*^−1^, one has to evaluate the one-electron contribution only. In the present paper, we evaluate this contribution to all orders in *Zα* for the 2*s*-state at *Z*=20 using the corresponding formula^22^. This result is combined with the radiative and second order in *m*_e_·*m*_nucl_^−1^ recoil corrections^19^,^21^,^23^,^24^ to get the total one-electron contribution. To evaluate the interelectronic-interaction contribution to the recoil effect of the first and higher orders in *Z*^−1^, we extrapolated the related results obtained to the lowest relativistic order^25^ (Methods section). The uncertainty of this contribution is mainly due to uncalculated higher order relativistic and QED corrections.

To get the total value of the isotope shift, one has also to account for the nuclear size effect. This contribution, being rather small, can be calculated in the one-electron approximation by solving the Dirac equation numerically. Moreover, it can be evaluated using an analytical formula^26^. The root-mean-square nuclear charge radii and their uncertainties are taken from ref. 7. The uncertainty of the nuclear size contribution includes both the nuclear radius and shape variation effects.

The individual contributions of the calculated isotope difference Δ*g*=*g*(^40^Ca^17+^)−*g*(^48^Ca^17+^) are presented in Table 1. It is seen that the QED recoil effect, whose calculation requires using QED beyond the Breit approximation and beyond the Furry picture, is about five times bigger than the total theoretical uncertainty.

### Measurement concept

For the experimental determination of the *g*-factor difference, we measured successively the Zeeman splitting of the respective lithium-like ion in a homogeneous magnetic field *B* using single ions confined in a Penning trap. The Larmor frequency *ν*_L_, which quantifies the energy difference between the spin-up and the spin-down state of the valence electron, is given by: 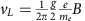. We determine the magnetic field by measuring the cyclotron frequency 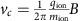 of the ion with electric charge *q*_ion_ and mass *m*_ion_. In the concluding equation for the *g*-factor:


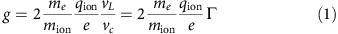


the magnetic field cancels, if in the ratio Γ≡*ν*_L_·*ν*_c_^−1^ both frequencies are probed simultaneously. To obtain the *g*-factor from the measured frequency ratios Γ, used in equation (1), the atomic masses of the ions are required. While the masses of ^40^Ca *m*(^40^Ca^17+^)=39.953272233 (22) u with a relative mass uncertainty of *δm*_ion_·*m*_ion_^−1^=0.6 parts per billion (p.p.b.; refs 27, 28) and also of the electron with *δm*_e_·*m*_e_^−1^=0.03 p.p.b. (ref. 9) are known with sufficient accuracy, the tabulated value of the mass of ^48^Ca is not adequately precise. In the following, we report on high-precision measurements of (i) the ^48^Ca mass and (ii) the frequency ratios Γ(^40^Ca^17+^) and Γ(^48^Ca^17+^).

### Determination of the atomic mass of ^48^Ca^17+^

With the Penning-trap mass spectrometer SHIPTRAP^11^, located at GSI Helmholtzzentrum für Schwerionenforschung Darmstadt, the atomic mass of ^48^Ca is directly determined by the measurement of the cyclotron-frequency ratio *R* of the mass doublet of singly charged ^48^Ca^+^ ions and ^12^C_4_^+^ carbon cluster ions: *R*≡*ν*_*c*_(^48^Ca^+^)/*ν*_*c*_(^12^C_4_^+^)=*m*(^12^C_4_^+^)/*m*(^48^Ca^+^). Instead of using the Brown–Gabrielse invariance theorem *ν*_c_^2^=*ν*_+_^2^+*ν*_z_^2^+*ν*_−_^2^ (ref. 29), both cyclotron frequencies have been determined as the sum of the ion's two radial eigenfrequencies *ν*_c_=*ν*_+_+*ν*_−_, where *ν*_+_ is the modified cyclotron frequency and *ν*_−_ the magnetron frequency. Considering a mass difference of Δ*m*=*m*(^12^C_4_^+^)−*m*(^48^Ca^+^)≈4.8 × 10^−2^ u we derive a systematic shift of the mass ratio Δ*R* <1 × 10^−11^ caused by possible misalignments and ellipticity of our trap. At the current level of precision, this effect is negligible.

**Figure 1:**
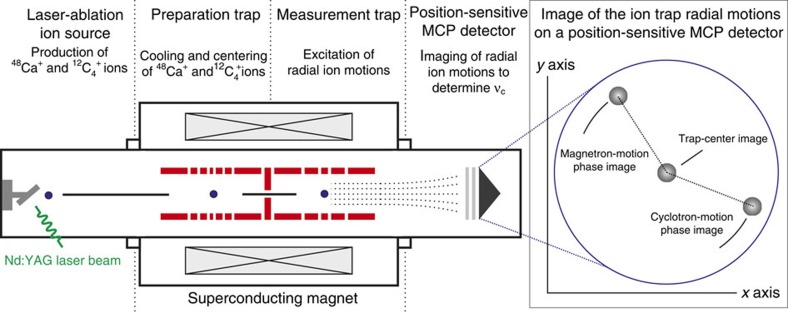
Offline configuration of the Penning-trap mass spectrometer SHIPTRAP. The set-up contains a laser-ablation ion source, two Penning traps, one for the preparation of the ion (cooling and centring), the other for the frequency measurement process and a position-sensitive multi-channel plate (MCP) detector for a radial resolution of the ion position. The novel PI-ICR is alternately applied to small clouds of ^48^Ca^+^ and ^12^C_4_^+^ ions, determining their respective cyclotron frequencies.

In each measurement cycle, we produce alternately small clouds (≤5 ions) of ^48^Ca^+^ and ^12^C_4_^+^ with a laser-ablation ion source^30^ and separately transfer them into a preparation trap for cooling and centring via mass-selective buffer-gas cooling^31^ (Fig. 1). Then, the particular cyclotron frequency is measured in the measurement trap with the novel PI-ICR (refs 12, 13; Methods section). Combining the measured cyclotron-frequency ratio *R*=1.00099010175 (35)_stat_ (17)_syst_ (*δR*·*R*^−1^=0.39 p.p.b.) with the known carbon cluster mass *m*(^12^C_4_^+^) and correcting for the missing electrons and their corresponding binding energies, we obtain the following value for the mass of lithium-like ^48^Ca (Methods section):


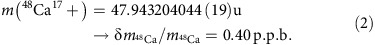


The resulting atomic mass agrees within its uncertainty with the previous less accurate measurements^32^,^33^.

### Measurement of the Larmor-to-cyclotron frequency ratios

**Figure 2:**
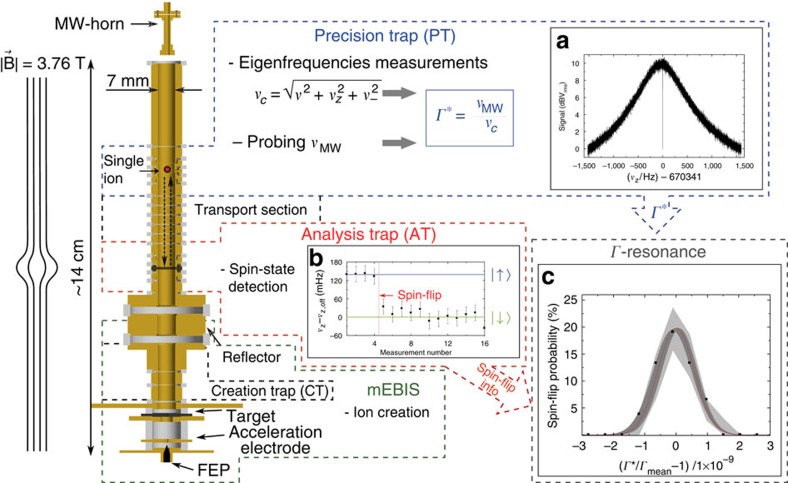
The *g*-factor experiment for highly charged ions. The triple Penning-trap set-up (gold) comprises: (i) The PT with a homogeneous magnetic field to determine the frequency ratios Γ* by measuring the three motional eigenfrequencies and probing the Larmor frequency. (ii) The AT to detect the spin-state of the valence electron. (iii) The Creation trap (CT) for ion creation within a miniature electron beam ion source (mEBIS). To enhance the production rate of ^48^Ca ions, an enriched calcium target is used with the following isotope composition: ^40^Ca: 78.77%, ^42^Ca: 3.02%, ^43^Ca: 0.62%, ^44^Ca: 9.55%, ^46^Ca: 0.02% and ^48^Ca: 8.02%. The set-up is placed in a cryogenic (*T*=4.2 K) ultra-high vacuum chamber (*P*<1 × 10^−16^ mbar). In **a** the axial resonator noise spectrum is shown including the dip-signal of a thermalized single ^48^Ca^17+^ ion. In **b** the spin-state of the ^48^Ca^17+^ ion is detected as an axial frequency jump at an absolute axial frequency of *ν*_z,off_=412.4 kHz. In **c** the spin-flip probability is shown in dependence of the measured Γ*-values, scaled by the final central Γ value Γ_mean_=5138.837 974 37 (58). The black points represent binned data to guide the eye. This data binning is not relevant for the Gaussian maximum-likelihood (ML) fit, shown in red. The dark grey-shaded area illustrates the uncertainty of Γ_mean_ and the bright grey area represents the binomial errors considering the amount of cycles of binned data and the probability of the ML fit. Error bars represent the uncertainties of each single axial frequency measurement point is related to the 1 sigma standard deviation.

Using a triple Penning trap set-up located at the University of Mainz, and described in detail in refs 34, 35, we measured the Larmor-to-cyclotron frequency ratio Γ of both calcium isotopes. Within a cryogenic (*T*=4.2 K) ultra-high vacuum chamber (*P*<10^−16^ mbar) a miniature electron beam ion source enables the production of highly charged ions. By means of various cleaning routines^35^ we remove all unwanted ion species and finally confine a single ion in a five electrode cylindrical Penning trap with an inner radius of *r*=3.5 mm. The oscillating ion induces image charges on the electrode surfaces, which we measure to obtain the axial oscillation frequency. In the attached superconducting, tuned axial resonator the induced oscillating currents generate a measureable voltage signal in the order of a few 10 nV. We detect the signal of the thermalized axial motion (*T*_z_∼5 K) as a minimum (‘dip-signal') in the Fourier transform of the thermal noise spectrum of the tank circuit (Fig. 2a). Both radial modes of the ion are thermalized and detected via rf-sideband coupling to the axial resonator generating double dip-signals in the axial frequency spectra. We determine the cyclotron frequency via the Brown–Gabrielse invariance theorem, where eigenfrequency shifts due to trap misalignment and ellipticity cancel^36^.

Simultaneously to the high-precision phase-sensitive measurement of the modified cyclotron frequency^37^, lasting about 5 s, we inject microwaves (MW) at the assumed Zeeman transition frequency (*ν*_MW_≈105 GHz) into the apparatus to induce spin-flips. To assess the success of a spin-flip attempt in our Precision trap (PT), we analyse the electron spin-state before and after the probing in a spatially separated Penning trap, the Analysis trap (AT). Here, a large magnetic bottle (*B*_*2,z*_=10(1)·10^3^ T m^−2^) couples the magnetic moment to the axial motion, resulting in frequency jumps of the axial oscillation 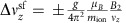, which are caused by changes of the electron's spin direction. This so-called continuous Stern–Gerlach effect^38^ enables the spin-state detection. In case of the ^48^Ca^17+^ ion 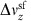 amounts to only 140 mHz at an absolute frequency of *ν*_z_=412.4 kHz, which represents a significant experimental challenge. Figure 2b illustrates the distinct detection of a spin-flip in the AT. Considering the limiting axial frequency resolution in the AT, we implement a proper cycle weighting to reduce the statistical uncertainty (Methods section).

During the automated measurement process, we probe the Zeeman transition several 100 times at different MW frequencies *ν*_MW_. Combining the corresponding measured frequency ratios Γ*=*ν*_MW_·*ν*_c_^−1^ with the binary information of the spin-flip, we obtain a Γ-resonance (Fig. 2c), which depicts the spin-flip probability in the PT versus the measured frequency ratios. With a weighted Gaussian maximum-likelihood fit, we extract the mean value Γ_mean_. This value has to be corrected for several systematic shifts (Methods section and ref. 39).

## Discussion

Combining the calcium masses with the measured frequency ratios Γ(^40^Ca^17+^)=4,282.42953545 (30) and Γ(^48^Ca^17+^)=5,138.83795612 (42), we derive the most precise *g*-factor values for lithium-like ions from equation (1):


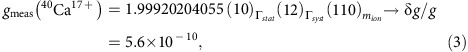



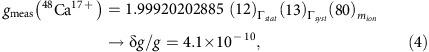


The statistical, systematic and ion mass uncertainties are given separately. The absolute values for the *g*-factors (Table 2) provide a stringent test of many-electron QED calculations in a magnetic field^40^,^41^. The *g*-factor difference finally yields the sought-after isotope difference:


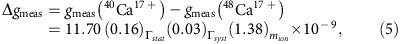


where the uncertainties of the frequency ratios and the mass measurements are listed separately. Obviously, the uncertainties in the masses of the isotopes dominate the total uncertainty. Since the dominant systematic shifts of the frequency ratios, the image charge shift (Table 3 and Methods section), scales with the mass of the ion, it cancels in the *g*-factor difference. Consequently, the denoted systematic uncertainty of the frequency ratios is smaller than the quadratically summed statistical uncertainties of the Γ-ratios given in equations (3) and (4). The comparison of the measured value of the *g*-factor difference with the theoretical prediction of this work:


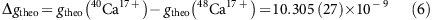


allows for the first time a direct test of the relativistic interaction of the electron spin with the motile nucleus. Although at present the experiment confirms the calculation only at the 10% level, the uncertainty of the measured frequency ratios is on the level of the QED recoil contribution.

Assuming QED calculations are correct within the given error bar, one may use the small uncertainty of the theoretically predicted *g*-factor difference in combination with the measured frequency ratios and the mass of ^48^Ca^17+^ to determine the isotopic mass difference: Δ*m*=*m*(^48^Ca)−*m*(^40^Ca)=7.9899317834 (54) u. The uncertainty of this indirectly obtained mass difference is a factor 5.7 smaller than the directly measured mass difference.

The combination of high-precision measurements of Larmor-to-cyclotron frequency ratios, atomic masses of the lithium-like isotopes ^40^Ca^17+^ and ^48^Ca^17+^ and corresponding *g*-factor calculations, presented in this paper, enables a variety of fundamental studies. Besides the test of many-electron QED calculations in a magnetic field by considering the absolute values of the *g*-factors or the indirect determination of the isotopic mass difference, the analysis of the measured and predicted *g*-factor difference between the calcium isotopes deepens the understanding of the interaction between the bound electrons and the nucleus. A further reduction of the mass uncertainties will enable an even more stringent test of the relativistic recoil predictions in the future. The validation of QED calculations is a prerequisite for further fundamental measurements in atomic physics, for example, the determination of the fine structure constant *α* via *g*-factor measurements of heavy, highly charged ions^14^.

## Methods

### Calculation of the isotope shift

The main contribution to the isotope shift Δ*g*=*g*(^40^Ca^17+^)–*g*(^48^Ca^17+^) results from the nuclear recoil effect that must be calculated including the relativistic, QED and interelectronic-interaction contributions. As the nuclear size effect is rather small, it can be evaluated in one-electron approximation by solving the Dirac equation.

Consider first the nuclear recoil effect on the atomic *g*-factor to zeroth order in 1/*Z*. In this approximation, the *m*_e_·*m*_nucl_^−1^ nuclear recoil contribution to the *g* factor of an ion with one electron over closed shells is given in refs 22, 23.


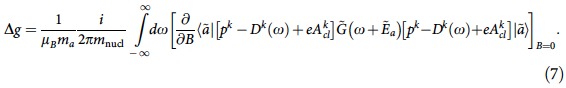


Here, *ℏ*=*c*=1, *e*<0, *μ*_*B*_ is the Bohr magneton, *m*_*a*_ is the angular momentum projection of the state *a*, *p*^*k*^=−*i*∇^*k*^ is the momentum operator, ***A***_*cl*_=[***B*** × *r*]/2 is the vector potential of the homogeneous magnetic field ***B*** directed along the *z* axis, *D*^*k*^(*ω*)=−4*παZα*^*l*^*D*^*lk*^(*ω*),


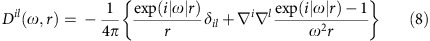


is the transverse part of the photon propagator in the Coulomb gauge. The tilde sign indicates that the related quantity (the wave function, the energy and the Coulomb-Green function 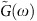) must be calculated in presence of the homogeneous magnetic field ***B*** directed along the *z* axis. As we consider an ion with one valence electron over the closed shells, the Coulomb-Green function is defined as 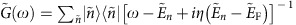, where 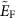 is the Fermi energy and *η*→0. In equation (7), the summation over the repeated indices (*k*=1,2,3), which enumerate components of the three-dimensional vectors, is implicit. Formula (7) incorporates both one- and two-electron nuclear recoil contributions to zeroth order in 1/*Z*. For the (1*s*)^2^2*s*-state of a lithium-like ion, the (1/*Z*)^0^ two-electron contribution is zero and, therefore, we restrict our consideration to the one-electron contribution only. For the practical calculations, the one-electron contribution is conveniently represented by a sum of low-order (‘non-QED') and higher order (‘QED') term, Δ*g=*Δ*g*_*L*_+Δ*g*_*H*_:


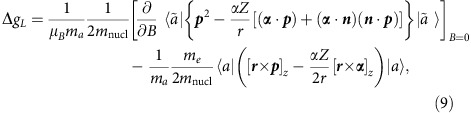



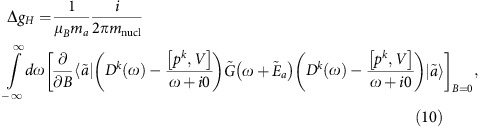


where *V*(*r*)=−(*αZ*)/(*r*) is the Coulomb potential induced by the nucleus and ***n***=***r**·r*^−1^. The low-order term can be derived from the relativistic Breit equation, while the derivation of the higher-order term requires using QED beyond the Breit approximation. For this reason, we call them the non-QED and QED contributions, respectively.

The low-order term Δ*g*_*L*_ can be evaluated analytically^42^:


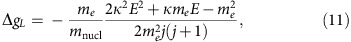


where *E* is the Dirac energy and 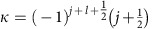. To the two lowest orders in *αZ*, we have


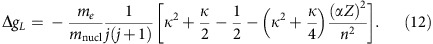


As follows from this formula, for an *s*-state (*κ*=−1) the non-relativistic contribution to Δ*g*_*L*_ vanishes and the low-order term comes from pure relativistic (∼(*αZ*)^2^) origin.

The calculation of the higher order term, Δ*g*_*H*_, is a much more difficult task. For the 1*s*-state it is calculated in ref. 42. In the present paper we performed the corresponding calculation for the 2*s*-state. Details of this calculation and the corresponding results for other ions will be published elsewhere.

In addition to the main one-electron nuclear recoil contribution, we have to consider the radiative (∼*α*) nuclear recoil correction and the (*m*_*e*_/*m*_nucl_)^2^ nuclear recoil correction. To the lowest order in *αZ*, these corrections were evaluated in refs 19, 21, 23, 24. We need also to account for the interelectronic-interaction effects of the first and higher orders in 1/*Z*. To evaluate these effects we extrapolate the lowest order relativistic results from ref. 25. The uncertainty of the interelectronic-interaction contribution is mainly due to uncalculated higher-order relativistic and QED corrections.

To get the total value of the isotope shift, we also evaluate the nuclear size correction. The root-mean-square nuclear charge radii and their uncertainties are taken from ref. 7. The uncertainty of the nuclear size contribution includes both the nuclear radius and shape variation effects. The individual contributions to the isotope shift of the *g*-factor for ^40^Ca^17+^ and ^48^Ca^17+^ are presented in Table 1.

In Table 2 we list the various contributions to the *g*-factor of ^40^Ca^17+^ and ^48^Ca^17+^. The Dirac value, as well as the QED, interelectronic-interaction, and the screened QED corrections^17^ cancel out in the isotope difference. The finite nuclear size and nuclear recoil corrections lead inherently to the isotope shift.

### The PI-ICR measurement scheme

After the transfer of the ions from the preparation trap into the centre of the measurement trap (Fig. 1), the coherent components of their magnetron and the axial motions are damped via 1 ms dipole rf-pulses at the corresponding motional frequencies to amplitudes of about 0.01 and 0.4 mm, respectively. These steps are required to reduce a possible shift in the ratio of the ^48^Ca^+^ and 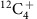
ions due to the anharmonicity of the trap potential and inhomogeneity of the magnetic field to a level well below 10^−10^ (see ref. 13 for details). After this preparatory step, the radius of the ion cyclotron motion is increased to a radius of 0.5 mm to set the initial phase of the cyclotron motion. Then, two excitation patterns, called in this work ‘magnetron-motion phase' and ‘cyclotron-motion phase', are applied alternately to measure the ion cyclotron frequency *ν*_*c*_. In the ‘magnetron-motion phase' pattern the cyclotron motion is first converted to the magnetron motion with the same radius. Then, the ions perform the magnetron motion for 100 ms accumulating a certain magnetron phase. After 100 ms have elapsed, the ions' position in the trap is projected onto a position-sensitive detector by ejecting the ions from the trap towards the detector^43^. In the ‘cyclotron-motion phase' pattern the ions first perform the cyclotron motion for 100 ms accumulating a certain cyclotron phase with a consecutive conversion to the magnetron motion and again projection of the ion position in the trap onto a position-sensitive detector. The angle between the ion-position images corresponding to two patterns with respect to the trap centre image is proportional to the ion cyclotron frequency *ν*_*c*_. Pulse patterns are applied for a total measurement time of ∼5 min. On this measurement scale the ‘magnetron-motion phase' and ‘cyclotron-motion phase' can be considered to be measured simultaneously. Data with >5 detected ions per cycle are not considered in the analysis to reduce a possible shift in the ratio of the ^48^Ca^+^ and ^12^C_4_^+^ ions due to ion–ion interaction. To eliminate a possible cyclotron-frequency shift, which arises due to incomplete damping of the coherent component of the magnetron motion, the time between the damping of the magnetron and axial motions and the excitation of the ion cyclotron motion is varied over the period of the magnetron motion. The positions of the magnetron motion and cyclotron motion phase spots are chosen such that the angle between the phase spots, calculated with respect to the centre of the measurement trap, do not exceed few degrees. This is required to reduce the shift in the ratio of the ^48^Ca^+^ and ^12^C_4_^+^ ion masses due to the possible distortion of the ion-motion projection onto the detector to a level well below 10^−10^ (ref. 13).

### Data sets for the ion cyclotron-frequency ratio *R*

**Figure 3:**
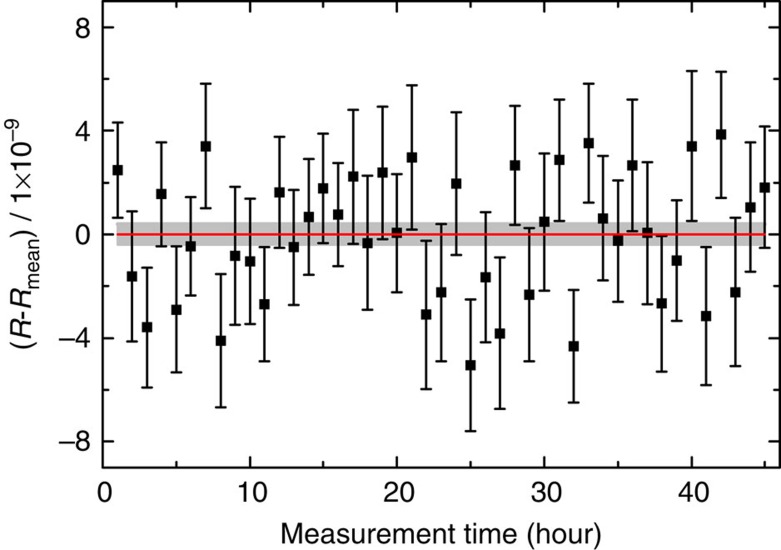
Data sets of the cyclotron-frequency ratio measurements *R*_1 h_ of ^48^Ca^+^ and ^12^C_4_^+^ at SHIPTRAP. The red line and the grey-shaded band illustrate the mean ratio *R*_mean_ and the s.d. For details on the plotted error bars see text.

The cyclotron frequencies *ν*_*c*_ of the ^48^Ca^+^ and ^12^C_4_^+^ ions are measured alternately for several days. The total measurement period is divided in 45 ∼1-h periods. In addition, each 5 min measurement is divided in 10 30-s periods. For each of the 45 1-h periods the ratio *R*_1 h_ of the cyclotron frequencies ^48^Ca^+^ and ^12^C_4_^+^ ions is obtained along with the inner and outer errors^44^ by fitting to the 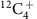 frequency points a polynomial of fifth order *P*_2_(t) with constant coefficients *a*_0_, *a*_1_, *a*_2_, *a*_3_, *a*_4_ and *a*_5_ and to the ^48^Ca^+^ frequency points a polynomial *P*_1_(t)=*R*_1 h_ × *P*_2_(t). The final cyclotron-frequency ratio *R*_mean_ is the weighted mean of the *R*_1 h_ ratios, where the maximum of the inner and outer errors of the *R*_1 h_ ratios are taken as the weights to calculate *R*_mean_ (Fig. 3). The difference between the inner and outer errors does not exceed 10%. The final frequency ratio *R* with its statistical and systematic uncertainties is *R*_mean_=1.00099010175 (35)_stat_ (17)_syst_. The systematic uncertainty in the frequency-ratio determination originates from the anharmonicity of the trap potential, the inhomogeneity of the magnetic field and the distortion of the ion-motion projection onto the detector^13^.

### The atomic mass of ^48^Ca^17+^

The mass of a C_4_^+^ cluster is calculated by considering the dissociation energy: E_diss_=18.0(17) eV (ref. 45), the ionization energy: E_ion_=11.0(7) eV (ref. 46) and the missing electron: 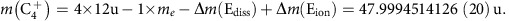 The mass differences between all three possible cluster structures—linear, rhombus and triangular pyramidal—are already covered by the uncertainties of the dissociation and ionization energies. For the determination of the mass of lithium-like ^48^Ca we have to correct the mass of singly charged ^48^Ca, *m*(^48^Ca^1+^)=*m*(C_4_^+^)/*R*, by the 16 missing electron masses and the corresponding ionization energies: Δm(*E*_bind_)=7.2438 (43) × 10^−6^ u, where *E*_bind_=6,747.5 (40) eV (ref. 47) and 1u=931,494,061 (21) eV c^−2^:


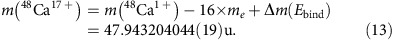


### The atomic mass of neutral ^48^Ca

For completeness, we also specify the atomic mass of neutral ^48^Ca. Correcting for the mass of the missing electron and its binding energy *E*_bind_=6.11315520 (25) eV (ref. 47) we obtain:


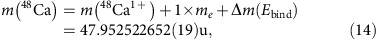


which is in good agreement with the literature value of *m*(^48^Ca)=47.952522765 (129) (ref. 28) but a factor seven more precise.

### Cycle weighting of the Γ-resonances

**Figure 4:**
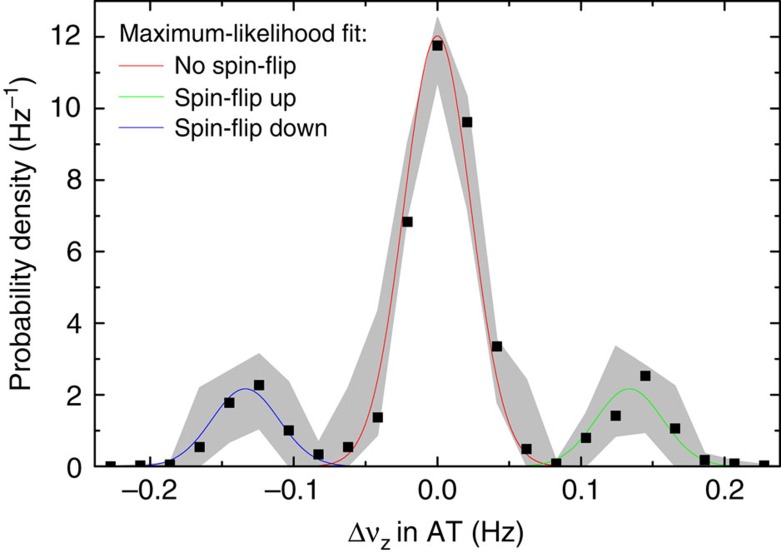
Probability density of the measured axial frequency differences in the AT. 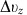
 is the axial frequency difference of subsequent measurements in the AT with 30 s spin-flip drives in between. From a maximum-likelihood (ML) fit, which combines three Gaussian distributions (red: no spin-flip (sf), green: spin-flip up, blue: spin-flip down), the following parameters are extracted: the spin-flip rate: 26.5%, the frequency jitter: 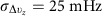 and the axial frequency jump due to a spin-flip: 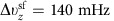.

In the magnetic bottle of the AT the axial frequency jump caused by an induced spin-flip scales with the inverse of the ion's mass. In contrast to our previous measurements, where the axial frequency shifts have been: 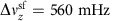 for ^12^C^5+^ (ref. 10), 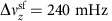 for ^28^Si^13+^ (ref. 9) and ^28^Si^11+^ (ref. 40), it is a particular challenge to resolve the spin-states for the calcium isotopes, where 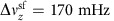 for ^40^Ca^17+^ and only 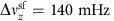 for ^48^Ca^17+^. We measure axial phase differences of subsequent measurements by applying a coherent detection technique, which includes three steps: (i) The axial phase is imprinted by a 10 ms dipolar excitation. (ii) The axial phase evolves for a certain time *T*_evol_. (iii) The phase is measured via the axial detection system. With a phase-evolution time of *T*_evol_=1 *s* and a readout-time of 552 ms, a spin-flip corresponds to an axial phase-shift of 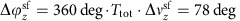 for ^40^Ca^17+^ and 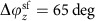 for ^48^Ca^17+^. In Fig. 4 1,790 averaged axial frequency differences of ^48^Ca^17+^ are histogrammed. Here, we determine each axial frequency by averaging over four successive phase measurements. Between these measurement sequences, we try to induce spin-flips for 30 s at maximum MW-power and at a fixed MW-frequency. The plotted probability density *ρ*_AT_ is modelled by a superposition of three Gaussian distributions:


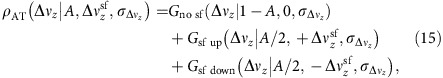


where *G*_no sf_ is the Gaussian distribution of the axial frequency differences without spin-flips with an amplitude (1-A), a mean value of zero and a s.d. of 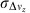. *G*_sf up_ and *G*_sf down_ are the Gaussian distributions with spin-flip up (mean value: 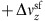) and spin-flip down (mean value: 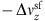). From a maximum-likelihood fit, the following three parameters are extracted: (I) the spin-flip rate: 26.5%, (II) the frequency jitter: 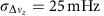 and (III) the axial frequency jump due to a spin-flip: 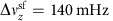. For the different data sets of ^48^Ca^17+^, we determine a frequency jitter of 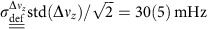. More precisely, we started with a tiny frequency jitter of 25 mHz and ended with a larger jitter of 35 mHz, although we optimized the trap harmonicity and checked the ion temperature. The reason of the declined frequency stability is unclear, but probably related to varying radiofrequency noise from external sources. As the largest measured jitter is only 2–2.9 times smaller than the cut-frequency difference of 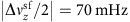 the probability of error of 0.5–4.5% is not negligible and has to be considered.

Instead of using a data analysis based on simple quality cuts, to decrease the probability of error and in that way losing statistics, we introduce the following AT-weight *w*_AT_ for each spin-flip, in a way that *w*_AT_=0, if the electron is in spin-down, *w*_AT_=1, if the electron is in spin-up and w_AT_=0.5, if the spin-state is unknown:


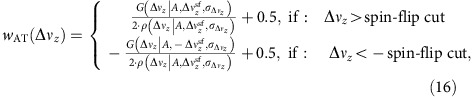


where the spin-flip cut is 70 mHz for ^48^Ca^17+^. In a normal measurement cycle, we try to induce a spin-flip at least three times in the AT and then proceed with this measurement process, until the cut-criterion |Δ*ν*_*z*_|> spin-flip cut is fulfilled for the first time. For the first and the last frequency jump in the AT, which fulfils this criterion, the AT-weight is calculated. The spin-flip probability in the PT (*w*_PT_) is calculated from the two AT-weights: before entering the PT 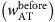 and directly after leaving the PT 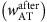:


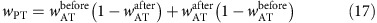


*w*_PT_=1 corresponds to a spin-flip in the PT, *w*_PT_=0 corresponds to no spin-flip in the PT and *w*_PT_=0.5 corresponds to no spin-flip information in the PT. The Gaussian line-shape of the Γ-resonance, which has been analysed in refs 9, 34, gets modified by adding a fourth fit-parameter (off_Γ_), which describes the wrong spin-flip detection rate in the PT:


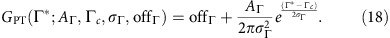


The PT-weight finally has to be included in the maximum-likelihood function:


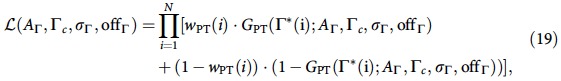


which is used, to extract the final mean value Γ_mean_. In comparison to the common cut-analysis, we improve the relative uncertainty of Γ_mean_ by 20 p.p.t.

### Data sets of the Γ-resonances

**Figure 5:**
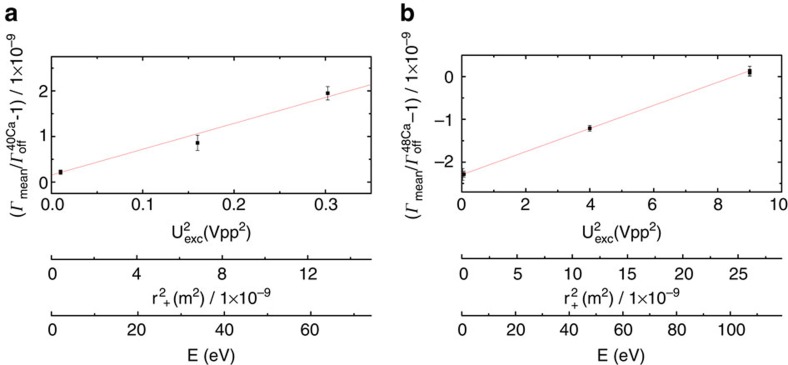
Γ-resonances at different modified cyclotron energies. We measure for both isotopes various Γ-resonances at different modified cyclotron energies. Here we show the extracted mean values Γ_mean_, normalized by the constant values: 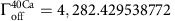 in **a** and 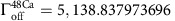 in **b** in dependence of the modified cyclotron energy, which is proportional to the squared amplitude U_exc_ of the first excitation pulse. The slope is mainly given by the relativistic mass shift in the cyclotron frequency. The indicated error bars illustrate the statistical uncertainty of the weighted maximum-likelihood fit (see the dark-shaded area in Fig. 2c).

Various Γ-resonances are recorded at different modified cyclotron energies during the phase-evolution time of the modified cyclotron mode and the simultaneous probing of the Larmor frequency *ν*_MW_ in the PT. In Fig. 5 the mean values from the maximum-likelihood fit, see equation (19), are plotted for ^40^Ca^17+^ (a) and ^48^Ca^17+^ (b). The slope is given mainly by the relativistic mass shift in the cyclotron frequency. The Larmor frequency is far less susceptible to relativistic shifts owing to the slow Thomas precession of the electron, which is bound to the heavy ion, leading to a suppression by a factor 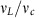. From linear extrapolations to zero modified cyclotron energy we derive our statistical Γ-values:


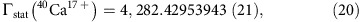



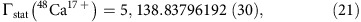


which have to be corrected by systematic shifts.

### Systematic shifts and uncertainties of Γ(^40^Ca^17+^) and Γ(^48^Ca^17+^)

The systematic shifts of the Larmor-to-cyclotron frequency ratios and the corresponding uncertainties are listed in Table 3. The dominant systematic shift and uncertainty is given by the image charge shift. Here, the induced image charges at the Penning trap electrode surfaces generate an additional effective electric potential, which shifts the radial eigenfrequencies of the ion. In ref. 39 the shift of the cyclotron frequency is analytically calculated:


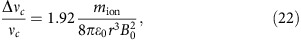


where *r* is the inner radius of the Penning trap. Due to the *r*^−3^-scaling, this shift can be reduced in future experiments by increasing the size of the Penning trap. All other systematic shifts, which are at least one order of magnitude smaller than the image charge shift, are explained in refs 10, 35.

## Additional information

**How to cite this article:** Köhler, F. *et al.* Isotope dependence of the Zeeman effect in lithium-like calcium. *Nat. Commun.* 7:10246 doi: 10.1038/ncomms10246 (2016).

## Figures and Tables

**Table 1:** Individual contributions of the calculated isotope difference Δ*g*=*g*(^40^Ca^17+^)−*g*(^48^Ca^17+^).

**Effects**	**Contributions to Δ*****g*****/1** × **10**^**−9**^
Nuclear recoil: one-electron non-QED∼*m*_e_/*m*_nucl_	12.246
Nuclear recoil: one-electron non-QED∼(*m*_e_/*m*_nucl_)^2^	−0.006
Nuclear recoil: one-electron QED∼*m*_e_/*m*_nucl_	0.123
Nuclear recoil: one-electron QED∼*α* (*m*_e_/*m*_nucl_)	−0.009 (1)
Nuclear recoil: interelectronic-interaction	−2.051 (25)
Finite nuclear size	0.004 (10)
Total theory	10.305 (27)

For details, see Methods section.

**Table 2:** Theoretical *g*-factor contributions for the lithium-like calcium ions ^40^Ca^17+^ and ^48^Ca^17+^.

**Effects**	***g*****(**^**40**^**Ca**^**17+**^**)**	***g*****(**^**48**^**Ca**^**17+**^**)**
Dirac value (point nucleus)	1.99642601090	
QED,∼*α*	0.002325555 (5)	
QED,∼*α*^2^	−0.000003520 (2)	
Interelectronic interaction	0.000454290 (9)	
Screened QED	−0.000000370 (7)	
Finite nuclear size	0.00000001441 (2)	0.00000001441 (2)
Nuclear recoil	0.00000006185 (15)	0.00000005154 (12)
Total theory	1.999202042 (13)	1.999202032 (13)
Measured *g*-factor	1.9992020405 (11)	1.99920202885 (82)

The Dirac value, as well as the QED, interelectronic-interaction and screened QED corrections cancel in the *g*-factor difference. The two predicted *g*-factors agree with the measured values.

**Table 3:** Systematic shifts and uncertainties of the Γ measurements.

**Effects**	^**40**^**Ca**^**17+**^ **(p.p.t.)**	^**48**^**Ca**^**17+**^ **(p.p.t.)**
Image charge shift	−941 (47)	−1130 (57)
Image current shift	11 (12)	−0.6 (10)
Magnetic field imperfections	0.46 (31)	0.45 (37)
Line-shape model of the dip-signal	0 (14)	0 (12)
Electric field imperfections	0.00 (39)	0.00 (51)
*ν*_−_measurement	0.0 (30)	0.0 (26)
Drift of axial potential	0.0 (12)	0.0 (12)
Relativistic shift	−0.010 (1)	−0.010 (1)
Line-shape model Γ resonance	0.0 (6)	0.0 (6)
Γ_stat_ from lin. extrapol. to zero E_+_	4,282.42953943 (21)	5,138.83796192 (30)
Γ (corrected for syst. shifts)	4,282.42953545 (21)_stat_ (22)_syst_	5,138.83795612 (30)_stat_ (30)_syst_

In the upper part the relative systematic shifts and their uncertainties are listed, which have to be added to the Γ_stat_ measurements to derive the final Γ values.
